# End of Life Virtual Reality Training: Medical Student Increased Empathic Ability

**DOI:** 10.1093/geroni/igab046.3012

**Published:** 2021-12-17

**Authors:** Marilyn Gugliucci, Daniel Manukhin, Elizabeth Dyer, Barbara Swartzlander

**Affiliations:** 1 University of New England College of Osteopathic Medicine, Kennebunk, Maine, United States; 2 University of New England, University of New England, Maine, United States

## Abstract

Introduction: It is unclear if medical student empathy declines by third year of clinical rotation trainings. Desensitization throughout the first two years may lead to decreases in empathy as a coping mechanism to avoid burnout in the clinical years. This study determined if self-assessed empathy increased after conducting an Embodied Labs, Inc. end of life virtual reality (VR) experience. Methods: Mixed methods, quantitative/qualitative, research were applied for University of New England (UNE) College of Osteopathic Medicine (COM) 2nd year medical students (N=174). They completed the 3-part 30 minute Clay Lab VR experience. UNE IRB approved pre/post-tests focused on empathy. Data were collected using RedCap. Closed questions were analyzed applying frequency analysis and paired-sample t-test through excel. Open-ended questions were analyzed through N-VIVO 12+. Results: The data included pre/post-tests from 146 students volunteers. Results indicated statistical significance (P=.01) in all closed questions except for question 7 (What is your view of conducting a full code on a patient with a DNR? (P=.14). The greatest difference seen between pre (23.97% agree or strongly agree) and post-test (64.38% agree or strongly agree) data was for question 3 (I gained knowledge about what hospice is by embodying Clay in this virtual reality lab); P= .00. Three qualitative themes included: Impact, Empathy, EOL Knowledge. Conclusion: This VR Lab experience increased self-assessed empathy at the time of Clay Lab completion; however, enduring empathy and learning about hospice/EOL has not been measured. Further research is suggested to determine the longitudinal impact of virtual reality education.

